# Big Data Analytics for Prostate Radiotherapy

**DOI:** 10.3389/fonc.2016.00149

**Published:** 2016-06-14

**Authors:** James Coates, Luis Souhami, Issam El Naqa

**Affiliations:** ^1^Department of Oncology, University of Oxford, Oxford, UK; ^2^Division of Radiation Oncology, McGill University Health Centre, Montreal, QC, Canada; ^3^Department of Radiation Oncology, University of Michigan, Ann Arbor, MI, USA

**Keywords:** radiotherapy, data mining, machine learning, big data, systems radiobiology

## Abstract

Radiation therapy is a first-line treatment option for localized prostate cancer and radiation-induced normal tissue damage are often the main limiting factor for modern radiotherapy regimens. Conversely, under-dosing of target volumes in an attempt to spare adjacent healthy tissues limits the likelihood of achieving local, long-term control. Thus, the ability to generate personalized data-driven risk profiles for radiotherapy outcomes would provide valuable prognostic information to help guide both clinicians and patients alike. Big data applied to radiation oncology promises to deliver better understanding of outcomes by harvesting and integrating heterogeneous data types, including patient-specific clinical parameters, treatment-related dose–volume metrics, and biological risk factors. When taken together, such variables make up the basis for a multi-dimensional space (the “*RadoncSpace”*) in which the presented modeling techniques search in order to identify significant predictors. Herein, we review outcome modeling and big data-mining techniques for both tumor control and radiotherapy-induced normal tissue effects. We apply many of the presented modeling approaches onto a cohort of hypofractionated prostate cancer patients taking into account different data types and a large heterogeneous mix of physical and biological parameters. Cross-validation techniques are also reviewed for the refinement of the proposed framework architecture and checking individual model performance. We conclude by considering advanced modeling techniques that borrow concepts from big data analytics, such as machine learning and artificial intelligence, before discussing the potential future impact of systems radiobiology approaches.

## Introduction

Prostate cancer is the second most common cancer among men and is the fourth most common cancer overall (1). In Europe alone, prostate cancer is the most commonly diagnosed cancer in men and accounts for approximately one-quarter of newly diagnosed cases per annum (2).

Fractionated radiation therapy (radiotherapy) is a primary treatment method for prostate cancer patients with localized disease – approximately one-quarter of patients have some form of radiotherapy incorporated into their treatment regimen (3). The widespread acceptance of radiotherapy as a first-line treatment modality can be attributed to high rates of local control and acceptable levels of normal tissue toxicity (4, 5).

Modern external beam radiation therapy (EBRT) delivery technologies, such as stereotactic body radiation therapy (SBRT) and volume-modulated arc therapy (VMAT), offer increased conformity and total dose while minimally damaging adjacent normal structures (6–8). These advanced treatment tools generate vastly more amounts of treatment-related data than contemporary counterparts, such as three-dimensional conformal radiation therapy (3D-CRT). In terms of outcomes analysis, this can render quantitative modeling of treatment plans and retrospective outcomes exploration more complicated.

Historically, dose–volume metrics alone were used in an attempt to explain aberrant toxicities or biochemical relapses (9). Canonical examples of this include either hot spots in overlapping regions between PTV and normal structures that were thought to independently induce adverse normal tissue effects or, conversely, suboptimal PTV coverage thought to be the main cause for inadequate local control (10). In recent years, however, it has been demonstrated that dose–volume metrics, while straightforward to obtain and contributing significantly to the analysis of radiotherapy outcomes, are not the only determining factors of success in predicting radiotherapy outcomes (11, 12). This has been shown in prospective application of dose–volume metrics whereby such metrics have proven to provide limited classification performance (13, 14). Aside from dose–volume data, the emergence of advanced imaging modalities and high-throughput “-omics” methods have led to the generation of enormous amounts of data that can similarly be used to predict outcomes.

The multi-dimensional space that EBRT-related biological, dosimetric and clinical variables span is referred to herein as the *RadoncSpace*. Two overarching predictive modeling approaches that exploit big datasets and search different sub-spaces of the RadoncSpace have surfaced in recent years: *radiomics* (use of imaging datasets for outcome prediction) (15) and *radiogenomics* (uncovering relationships between biological data and outcomes) (16). Pioneering application of these two techniques speaks to the ever-increasing application of data-mining techniques and big data analytics (the so-called “*panomics”*) to modern oncology (4).

The specific objective of big data analytics in radiotherapy is to develop predictive models that capture underlying factors contributing to the development of selected endpoints without over-fitting noise or under-fitting trends. In line with the nature of big data and the heterogeneity of patient populations, a strict requirement of such modeling frameworks is that input datasets must be large enough to include variability, which accurately reflects the underlying patient population. Otherwise, resulting models can suffer from poor prospective prediction performance.

Clinically, it has already been demonstrated how such models could be used to better inform patients of treatment-associated risks. Namely, by integrating outcome models into treatment planning systems (TPSs) and recommending dose-escalation or dose-reduction (11). Given the potential future impact of outcome models in the clinic, the selection of tools and models for the fabrication of a predictive framework must be chosen carefully in a way to facilitate identification of optimal models.

In this work, we focus our attention exclusively on outcomes associated with EBRT; however, the presented modeling techniques are easily generalizable to any dose distribution. In this work, we briefly review the radiobiology of prostate cancer as a basis for understanding the theoretical underpinnings of analytical outcome models. Analytical models attempt to predict radiation-related toxicities by formalizing abridged versions of the biological processes by which selected endpoints become manifest. Subsequently, we discuss big data and data-driven modeling approaches based on techniques previously used successfully for exploring outcomes in radiotherapy. In contrast to analytical models, data-driven models are entirely empirical in nature, potentially making them more robust albeit more difficult to analyze or interpret. We then consider techniques that optimize model parameters in order to maximize model robustness and prevent under-/over-fitting, which are two common pitfalls in big data outcome modeling. The article concludes by presenting modeling techniques based on advanced artificial intelligence as well as on systems theory.

## Prostate Cancer

### Pathology

Adenocarcinoma of the prostate is the most common histopathological type of prostate malignancy and typically arises in the peripheral zone (17). Up to one-half of men present with prostate cancer at time of autopsy although tumors identified in many of these cases are typically small, impalpable, and of low grade (18).

Prostate tumors are known to have remarkable biological heterogeneity from patient to patient and even across tumor volumes (19–21). The metastatic potential of prostate cancer is similarly variable and is furthermore reflected in the wide variation in overall survival rates for those with localized disease at time of diagnosis (21). Notably, the high degree of heterogeneity makes standardization and characterization of prostate adenocarcinoma phenotypes challenging and institution specific.

### Basic Radiobiology

The α/β originates from the linear-quadratic (LQ) formulation of *in vitro* cellular survival experiments (Eq. 1):

(1) $$\mathrm{SF}=e^{-\left(\alpha D+{\beta D}^{2}\right)}$$

where SF is the surviving fraction after a dose (D). The coefficient α [Gy^−1^] in front of the linear dose term (D) relates to single-hit inactivation and the β [Gy^−2^] coefficient pertains to the expected rate of double-hit (two-track) cellular inactivation (22, 23). The α/β ratio taken from the LQ model allowed numerous fundamental radiobiological questions to be answered quantitatively. It remains a relevant parameter in radiotherapy today due to its clinical significance as a measure of tissue-specific fractionation-sensitivity.

It is well known that prostate cancers are relatively slow-growing malignancies with low α/β ratios (24–27), unlike most malignancies. When used in the context of biologically effective dose (BED) (Eq. 2), the low prostate α/β ratio (~1.5 Gy) translates to a high sensitivity to fraction size:

(2) $$\text{BED}=D\cdot \left(1+\frac{d}{\alpha /\beta }\right)$$

where *D* is the total dose of the radiotherapy regimen and *d* is the fraction size. Furthermore, since the α/β ratio for prostate cancer is lower than that of normal tissues (~3 Gy), an improved therapeutic ratio can be expected using hypofractionation (28–30).

In 2013, Vogelius and Bentzen performed a meta-analysis of 1965 patients derived from five separate studies (31). In line with pioneering work by Fowler and colleagues (25), they showed that prostate cancers do indeed have an exceedingly low α/β ratio. Interestingly, after accounting for changes during treatment, their estimate of α/β increased. This may indicate that the α/β for prostate tumors changes throughout the course of radiotherapy treatment, probably due to subpopulation selected induced by radiotherapy itself.

Although α/β has provided insight into radiobiology of prostate cancer, it remains unclear how relevant the ratio is in cases of modern EBRT delivery, such as high-dose hypofractionated SBRT regimens, mixed-modality treatments (photon with proton boost) or when using charged particles, such as carbon ion, all of which are becoming increasingly popular treatment options for prostate cancer. In such cases, aggregation of large-scale datasets serving as inputs to big data analytics may provide more useful insight either as a supplement or as a substitute to classical paradigms in radiobiological modeling.

## Types of Outcomes

Toxicity outcomes in radiotherapy can be segregated into two categories: acute (effects observed within 3 months after the termination of radiotherapy) and late (effects that manifest after the 90-day cutoff). Furthermore, normal tissue damage can be segregated by site; in prostate radiotherapy, normal tissue side effects manifest themselves most frequently as one or more of gastrointestinal (GI) toxicities, genitourinary (GU) toxicities, or erectile dysfunction (ED).

### Acute (Early) Outcomes

Acute effects due to normal tissue damage from ionizing radiation in prostate cancer radiotherapy regimens include GI/GU symptoms. Acute symptoms are most often transient, self-limiting events in that they appear and resolve within a matter of weeks without contributing significantly to severe or long-term morbidity, although some consequential late effects in prostate radiotherapy have indeed been recorded (32–34).

A 2015 review article by Drodge et al. compiled the results of 22 prospective hypofractionated trials completed between 2001 and 2013 (35). Using the RTOG/EORTC toxicity grading scheme and including studies that used different treatment modalities and schedules, the authors concluded that Grade 3 acute toxicities, on the whole, affect less than 10% of prostate patient cohorts receiving hypofractionated EBRT. Furthermore, Grade 2 toxicities affected under half of patients. The study also expanded upon the practical challenges in interpreting outcome data from independent trials that may use different grading schemes or endpoints.

Proton therapy is becoming more common in modern times for use in treating prostate cancer (36). Studies have shown that the frequency of acute effects with proton for prostate cancer is not significantly increased over that of conventionally fractionated photon therapy regimens (37, 38). One dose-escalation study with 85 prostate cancer patients using proton doses up to 82 Gy-equivalent (GyE) yielded acute toxicity levels comparable to photon radiation (39).

### Late Normal Tissue Endpoints

Radiation-induced late normal tissue damage consists of toxicities that occur >90 days after completion of radiotherapy. Late toxicities can range from mild, moderate, severe to life-threatening requiring immediate intervention. They are categorized as either GI or GU effects and many retrospective studies report only these outcomes; however, sexual dysfunction is also considered herein. Unfortunately, the full pathophysiology of the radiation-induced manifestation of ED has yet to be fully elucidated.

The difficulty in assessing late toxicities is that often times no quantitative physiological evidence exists or can readily be obtained. Grading schemes have been developed to resolve such issues. Grading schemes require physicians to assign integer values to the radiation-induced side effect based on selected criteria. Some schemes utilize self-scoring questionnaires, while others rely on grades assigned by attending oncologists. Interestingly, several groups have sought to explore the correlation between different scoring schemes using a single set of data in order to explore what role grading schemes have on incidence rates of toxicity (40–42). Such works bear significance for the use of big data analytics as many frameworks utilize supervised learning techniques that rely on the accuracy of outcome measures.

### Local Control Endpoints

It is estimated that overall approximately one-third of prostate cancer patients experience some type of biochemical relapse within the first decade after completion of their EBRT treatment regimen (43). In reporting local control outcomes, clinical studies typically do so according to specific criteria, such as the ASTRO-RTOG Phoenix definition of local biochemical failure (44). Guidelines often include prostate serum antigen (PSA) scores although derivatives of simple PSA scores have also been considered, such as PSA doubling time (43). However, it should be noted that a rising PSA does not always indicate a local failure and it can antedate the diagnosis of metastatic disease for many years; thus, caution should be exercised when using it as a surrogate for local failure endpoint.

## Data Types

### Dose–Volume Metrics

Typical dose–volume metrics used in outcomes modeling include dose to a given volume or volume of tissue receiving at least a particular dose. These parameters can be readily extracted from dose–volume histograms at the treatment planning stage. Physiological changes, such as weight gain/loss or changes in tumor composition or anatomical position, may take place during treatment and, thus, dose delivered may not necessarily reflect biologically absorbed dose. It is likely that dose–volume variables could have their predictive accuracy improved by incorporating intra-fractional computed tomography (CT) scan changes, as has been considered in literature (45).

The equivalent uniform dose (EUD) (46) is a dose–volume metric that can be used to describe inhomogeneous dose distributions. The generalized EUD (gEUD) (47) is a further extension used for normal tissues of interest (Eq. 3):

(3) $$\text{gEUD}={\left(\sum_{i}v_{i}D_{i}^{a}\right)}^{1/a}$$

where the variables *v_i_* is fractional volume for the tissue exposed to dose *D_i_*, the parameter *a* is a factor relating to the volume effect of a given tissue type. These two metrics appear oftentimes in analytical outcome models as they serve as excellent tools to summarize dose distributions across volumes.

### Clinical Parameters

Clinical data can be parameterized and used to investigate covariates of interest. An example in the case of prostate cancer is in patients receiving anti-coagulant therapy and presenting with rectal bleeding (RB) late in their follow-up period, which can otherwise easily be mistaken for a late toxicity. Another case is the combined use of androgen deprivation therapy (ADT) since ED can be a side effect of ADT alone and thereby lead to an increased prevalence of late ED in a given prostate patient cohort.

### Spatial Parameters

Spatial dose–volume histograms (zDVHs) can be used to compare spatial treatment planning information to outcomes (48–50). The advantage of incorporating spatial information is that it provides modeling frameworks information about the location of dose extremes and, thus, mitigates having to rely solely on approaches based on volume-averages (or gEUD). This reduces the risk of under- or over-valuing the contribution of hot or cold spots. Spatial data can also provide information related to the contribution of hot spots in accessory structures, for example, in the case of rectal contour overlap with the PTV.

### Biological Variables

Several types of biological variables have been used previously in attempting to elucidate mechanisms by which prostate radiotherapy toxicities become manifest. The most popular class of variables found in literature today is related to genetic mutations. Additionally, work has been performed on exploring the role of epigenetics (51) and transcript expression levels (52) in long-term radiotherapy outcomes.

#### Genetic Variables

Given the relatively disappointing prospective predictive power of singular classes of genetic variables on their own (53–56), it is likely that modeling frameworks will need to allow for several types to be incorporated in a given model in order to maximize prospective classification performance.

Single-nucleotide polymorphisms (SNPs) consist of single-nucleotide changes. Their presence in certain genes or regulatory regions has been shown to be well correlated with prostate radiotherapy-related outcomes (52, 54, 57, 58). This is probably due to altering functional transcripts or protein confirmations after translation.

Further to SNPs, copy number variations (CNVs) have recently been of increasing interest to the radiotherapy community (59). CNVs reflect the number of copies of a particular gene and are, therefore, larger structural genetic mutations than SNPs. This could mean that larger changes in a given genome could be seen with CNV changes.

In our previous work, we have shown the value of integrating CNVs alongside SNPs (of the same gene) together with dose–volume metrics (60). Specifically, we have demonstrated that changes in the gene concentration of DNA repair gene XRCC2 can predict severe (Grade 3) late RB for hypofractionated prostate patients treated with 3D-CRT. More importantly, the resulting radiogenomic models led to increased predictive power as compared to using either type of genetic variable alone. We, furthermore, demonstrated that the improvement using SNPs and CNVs is not limited to data-driven frameworks but could also be applied to analytical models. These results indicate that different genetic mutations in the same gene may contribute similarly to a given outcome. If proven to be the case, it is likely a result of outcome scores being limited snapshots of complex pathophysiological events reflecting more than one biological alteration.

##### Integrating Genetic Variables in Outcome Models

In the case of data-driven modeling, genetic parameters can be considered as independent variables and regressed alongside clinical risk factors and dose–volume metrics. For analytical models, the method in which genetic parameters are integrated depends on the nature of the model at hand. In 2006, two groups showcased how dose-modifying factors (DMFs) extracted from clinical risk factors could be used to stratify standard analytical models and thereby generate “mixed” data-type models (57, 61). In 2013, Tucker et al. expanded this approach to include SNPs using an approach easily generalizable to any biological variable and demonstrated significantly improved classification performance (62). Rancati and colleagues further extended this approach using clinical risk factors for Logit and EUD models (63), from which our group drew inspiration in developing radiogenomic models using biological, clinical, and dosimetric variables (60).

#### Other Biological Variables

##### Epigenetics

The importance of epigenetic alterations to the genetic code has not by any means been understated by the scientific community in recent years (64–67). However, the significance of epigenetic modifications in radiotherapy remains to be fully understood. Research related to epigenetics and radiotherapy could be complicated by the fact that mounting evidence implies that radiotherapy itself can induce epigenetic changes (67).

Thus far, thousands of differentially methylated regulators have been identified in many cancer types thanks to epigenome-wide association studies (EWAS) (68). Differentially regulated promoter may serve as novel biomarkers to predict risk of biochemical relapse or serve as indicators of normal tissue radiosensitivity. In prostate cancer specifically, wide-ranging hypo- and hyper-methylations have been identified that correlated with early-stage carcinogenesis and aggressive tumor phenotypes (69, 70). Efforts are underway to generate an epigenetic code (71–73), which may facilitate the ability to perform and interpret EWAS results as well as provide a new class of input data for outcome models (74).

##### High-throughput Proteomics and mRNA Expression Levels

Numerous methods used to quantify large numbers of biological factors have been pioneered and introduced into mainstream biology research within the last decade. These technologies include well-characterized microarrays and proteomic analysis technologies that can quantify the levels of expression of up to tens of thousands mRNA transcripts or proteins in a single sample.

After generating large quantities of data, high-throughput modeling frameworks can be used that are able to deal with large numbers of variables (75). This approach has been used successfully in clinical oncology to stratify tumor phenotypes and estimate prognoses to help guide optimal therapeutic regimens (76–78). In the case of radiation oncology, high-throughput data have yielded several multi-gene signatures for hypoxia (79–81).

The challenges of utilizing a large number of variables in outcome models are well summarized by the multiple testing dilemma: too few samples relative to a large number of variables being tested can lead to spurious correlations. Even after utilizing simple supervised learning algorithms to pre-process the data, the number of mRNA transcripts that a single microarray experiment can yield is often in the thousands (82). This issue can be mitigated by large-scale validation studies but these are expensive, time-consuming and patient accrual can limit achieving the necessary sample size.

Alternatively, methods in artificial intelligence are becoming increasingly popular to explore the complex, hidden relationships between outcomes and biological variables (83). In contrast to brute-force estimating of correlations, machine-learning techniques in artificial intelligence have the ability to process highly structured, high-dimensional data while controlling for over- and under-fitting by drawing on methods from control, probability, and information theory.

## Modeling Techniques

### Risk Quantification

The likelihood of obtaining local control is quantified mathematically by tumor control probability (TCP). TCP is a probability that indicates chances for success of a treatment according to a particular endpoint, usually long-term control. Many studies using TCP-based approaches have shown that cancer cells *in situ* have complex, high-dimensional repopulation kinetics when exposed to ionizing radiation and/or chemotherapy (84–87). Such kinetics can lead to complex models and be dependent upon several factors, such as repair capacity, quality of radiation, fractionation scheme, and surrounding microenvironment (88).

In the case of normal tissue side effects from radiotherapy, risk is quantified via normal tissue complication probability (NTCP). NTCP values can be tailored to each individual treatment regimen to reflect the risk of a given side effect. Conventionally, such frameworks were limited to dosimetric data; however, it is now understood that late normal tissue toxicities are furthermore functions of a variety of biological, physical, and clinical factors (16, 89).

### Analytical Modeling

As previously described, models of the analytical class are based on simplified theoretical mechanisms of action radiobiological interactions. They include some level of mechanistic insight into a specific mechanism by which radiotherapy outcomes become manifest and are, therefore, also referred to as *mechanistic models*.

#### Tumor Control Probability

Cells that can lead to tumor growth are termed tumorigenic stem cells or cancer stem cells. These cells are, in theory, the primary targets of anti-cancer therapies. The probability that a given treatment will induce eradication of cancer stem cells for a given patient is mathematically given by the TCP.

##### The Linear-Quadratic

The LQ model has gained popularity in literature since it follows survival closely at conventional doses of radiation. Furthermore, the model provides a simplified theoretical basis for how radiation induces cellular deactivation: radiation tracks interacting with DNA can induce severe damage on its own (α component) or can combine with another track to increase density of damage (β component).

Questions have, however, arisen to the relevance of the LQ model for more modern treatment regimens, such as SBRT or charged particle therapy. It has been shown that the LQ model begins to deviate significantly from experimental data beginning at or around 6–8 GyE (22, 90, 91). Practically speaking, this does not affect conventional treatment regimens utilizing 2–3 Gy fraction sizes; however, the LQ model may predict effects of hypofractionation regimes poorly. One such example is the carbon ion lung trial in Japan whereby single fractions of 50 GyE were delivered (92). Furthermore, when considering cases *in vivo*, the standard LQ model does not take into account repopulation kinetics of cancer cells during intra-fraction periods, rendering it approximate at best (93).

##### Modified LQ Models

Modified versions of the canonical LQ model have been proposed to address some of the aforementioned shortcomings. Examples include generalized versions that have been further parameterized to account for repopulation (94), mixed radiation qualities (95), tumor heterogeneity, arbitrary or variable dose-rates (91), cell death mechanisms (96), and others able to take into account more than one of the aforementioned parameterizations (97).

In the case of charged particle therapy, the theory of dual radiation action (TDRA) predicts an increased linear component of LQ-modeled cell kill (α) over the quadratic component (β) (98). Indeed, it has been shown that β remains relatively stable in comparison to the variation of α across linear energy transfers (LETs) (99). Consequently, by considering the relative biological effectiveness (RBE) between a given high LET radiations and clinical energy photons, TDRA predicts that RBE will reach a minimum (RBE_min_) at very high doses, while at low doses RBE will reach an intrinsic maximum (RBE_max_) (100). In practice, it has been observed that both parameters α and β vary with LET. Thus, a modified LQ model has emerged that RBE_min_ and RBE_max_ are taken into account by further parameterizing the high-LET α and β values (Eq. 4a,b).

(4a) $$\alpha_{\text{H}}=\alpha_{L}\cdot {\text{RBE}}_{\max}$$

(4b) $$\beta_{H}=\beta_{L}\cdot {\text{RBE}}_{\min}^{2}$$

where α_H_ and α_L_ refer to α components of the high and low LET radiations, respectively, and β_H_ and β_L_ refer to the quadratic components of the high and low LET radiations, respectively.

In considering high-dose per fraction, such as those delivered by a SBRT prostate radiotherapy plan or charged particle therapy, the modified LQ model first proposed by Sachs et al. in 1997 (101) and extended in 2004 by Guerrero and Li (91) has been demonstrated to fit survival data well (Eq. 5a,b).

(5a) $$\mathrm{SF}=e^{-\alpha D-\beta \cdot G\left(\lambda T\right)\cdot D_{2}}$$

(5b) $$G\left(\lambda T\right)=2\left(\lambda T+e^{-\lambda T}-1\right)/{\left(\lambda T\right)}^{2}$$

where λ is the repair rate and T is treatment delivery time while the other parameters are taken from the standard LQ model. G(λT) is the dose protraction factor that specifies the contribution of misrepair to lethality (which is reduced at high, acute doses). Using this formulation of the LQ model, the large differences in cellular survival observed between predicted and experimental data are practically eliminated up to doses of ~30 GyE (91).

Review articles by Jones and Dale (23) and Zaider and Hanin (102) have elegantly summarized and discussed additional models and can be consulted for further details.

#### Normal Tissue Complication Probability

The objective of NTCP models is to gage the risk of inducing particular normal tissue effects, such as severe RB in the case of late prostate radiotherapy. A plethora of modeling techniques have been proposed for such purposes (103–105), some of which are described in more detail below.

##### Lyman–Kutcher–Burman

The most readily applied analytical method for generating NTCP values is through the Lyman–Kutcher–Burman (LKB) approach (106) (Eq. 6a,b).

(6a) $$\text{NTCP}\left(D,D_{50},m\right)=\frac{1}{\sqrt{2\pi }}\int_{-\infty }^{t}e^{\left(-\frac{u^{2}}{2}\right)}\mathrm{du}$$

(6b) $$\text{where }t=\frac{\text{EUD}-{\text{TD}}_{50}}{m\cdot {\text{TD}}_{50}}\text{ and }{\text{TD}}_{\text{50}}\left(V\right)=\frac{{\text{TD}}_{50}\left(1\right)}{V^{n}}$$

where *m* is the slope of the best-fit NTCP sigmoid, *TD_50_(1)* is the dose at which NTCP = 50% for a specific endpoint, and *TD_50_(V)* is the tolerance dose for a given partial volume with tissue-specific volume exponent *n*. Simply put, the LKB model stratifies patient risk according to how much larger or smaller their EUD is relative to the TD_50_. The EUD is a three-dimensional DVH reduction technique according to (Eq. 7).

(7) $$\text{EUD}={\left(\sum_{i}v_{i}\cdot D_{i}^{\frac{1}{a}}\right)}^{a}$$

where parameter *a* is 1/*n*, and *D_i_* and *v_i_* are the dose and partial volume, respectively, according to each DVH segment *i*. Expansions and more intricate variations of the canonical LKB model can be found readily throughout literature (57, 60, 62).

##### Binomial Models

*The Critical Volume* Functional subunits (FSUs) are thought to be fundamentally underlying structured subunits housing numerous cells in a given tissue. Perhaps the most readily available example is the crypt subunit in the GI tract that together forms the organ. FSUs have varying properties, shapes, and sizes and are tissue type specific. Such variation can be exploited by the critical volume (CV) model (Eq. 8) to account for the differences in radiation response between different tissue types (107).

(8) $$P_{t}=\left(\begin{array}{c} N\\ t \end{array}\right)P_{\mathrm{FSU}}^{t}{\left(1-P_{\mathrm{FSU}}\right)}^{N-t}$$

where the first term is the binomial coefficient of *N* and *t*. P_FSU_ is the probability that *t* of *N* subunits will be deactivated by ionizing radiation. Accordingly, the chance of *M* or more subunits being deactivated in a single exposure can be calculated according to:

(9) $$P=\sum_{t=M+1}^{N}P_{t}=\sum_{t=M+1}^{N}\left(\begin{array}{c} N\\ t \end{array}\right)P_{\mathrm{FSU}}^{t}{\left(1-P_{\mathrm{FSU}}\right)}^{N-t}.$$

**Figure 1:**
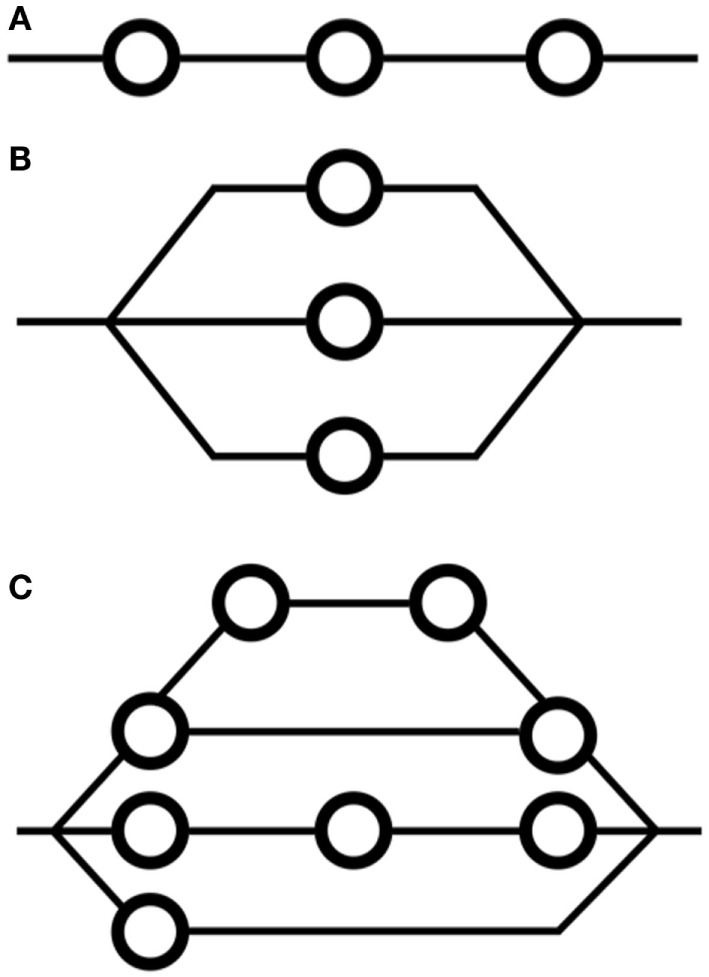
**Arrangements of functional subunits in (A) serial formation, (B) parallel formation, and (C) complex, or mixed, formation**. FSUs are functional compartments of a given organ. The concept of FSUs underpins many models for modeling NTCP, including the critical volume and relative seriality models.

Two major classes of tissue exist in the context of FSUs (Figures 1A,B): serial and parallel. Organs that are serial can have their function compromised by exposure of a limited volume (a “*critical volume*”) to a given dose, e.g., colon, spinal cord, brain stem. Note that the output of a serial organ is not a sum of its internal components as it is in the case of parallel organs. Thus, for parallel-type tissues, catastrophic damage to one part of the underlying physiological architecture does not risk the collapse of the organ itself, e.g., liver, skin. In reality, every tissue has a mix of both serial and parallel structures, a concept referred to “complex” FSU arrangement (Figure 1C), although some tissues are more one type than the other.

Interestingly, it has been shown that the LKB model can be derived upon reformulation of the CV model. This implies that the LKB model has a basis relating to FSUs (108).

Prior to mainstream applications of data-driven modeling techniques, modifications to the CV model (109, 110) and extensions of the FSU concept (111) were shown to be useful. Applications of the CV model in prostate cancer to predict late endpoints relating to bladder, colon, bowel, penile bulb, and rectum have been performed; however, their usefulness in practice has been limited compared to models identified using contemporary data-driven modeling approaches (112, 113).

*Relative Seriality* The relative seriality model for NTCP modeling was developed in order to consider and exploit arbitrary combinations of serial and parallel FSU arrangements (114). In such cases, risk of normal tissue damage is given by the following equation:

(10) $$\text{NTCP}\left(D,V\right)={\left[1-{\left(1-P{\left(D\right)}^{S}\right)}^{V/\mathrm{Vref}}\right]}^{1/s}$$

where the exponent V/V_ref_ is the fraction of volume that is being irradiated to the given dose, *D*, and the parameter *S* relates to the degree of seriality of the organ at risk – nearly 0 in the case of highly parallel structures and higher for mixed or serial structures. The function *P(D)* is the value of risk and can be derived via Poisson statistics:

(11) $$P\left(D\right)=e^{-N_{o}\cdot S\left(D\right)}$$

where *N_o_* is the number of FSUs and the function *S*(*D*) is the probability of a given FSU in the order of interest to survive irradiation to dose *D*.

### Data-Driven Modeling

Data-driven approaches to modeling are often referred to as phenomenological or statistical techniques. Models generated by such frameworks are based on empirical combinations of observations and are, thus, generally more robust than their analytical counterparts.

When considering dose–volume metrics alongside clinical and genetic risk factors, the number of variables can quickly become overwhelming and so data-driven modeling frameworks often include steps that seek to optimize and pre-process input data.

The most frequently employed approaches to data-driven modeling in radiotherapy are regression-based techniques. Regression link functions are typically chosen to be sigmoidal in order to achieve the non-linear dose–responses seen experimentally. Advanced methods in artificial intelligence that are able to handle non-linear data more readily are discussed later in this section. Such methods are becoming increasingly popular due to superior prospective classification performance in many areas of oncology (15, 115, 116).

Several review articles discussing the shortcomings and advantages of data-driven modeling can be found elsewhere in the literature (11, 117, 118).

#### Probit- and Logit-Based Regression

Link functions can be used in tandem with regression frameworks to fit either TCP or NTCP data. The use of an inverse-logit (Eq. 12) or -probit (Eq. 13) are examples of such functions.

(12) $$\pi \left(X_{i}\right)=\Phi \left(g\left(X_{i}\right)\right)$$

(13) $$\pi \left(X_{i}\right)=\frac{e^{g\left(X_{i}\right)}}{1+e^{g\left(X_{i}\right)}}=\frac{1}{1+e^{-g\left(X_{i}\right)}}$$

where g(x_i_) is the generalized linear model (GLM) formulation of the input variables: x_i_:

(14) $$g\left(X_{i}\right)=\beta_{0}+\sum_{j=1}^{s}\beta_{j}X_{\mathrm{ij}},i=1,\ldots ,n,j=1,\ldots ,s$$

where β coefficients are estimated according to maximum likelihood estimation (MLE).

Historically, the logit function has been employed more often than the probit function because of ease of use and mathematical simplicity.

In terms of interpretation in the context of prostate radiotherapy, data-driven models have the added benefit of being able to handle multiple types of data while independently stratifying the contribution of specific variables. This can again be contrasted with analytical models wherein parameters need only be estimated rather than having to entirely develop the model itself.

#### Artificial Intelligence (Machine Learning)

Techniques in artificial intelligence applied to outcome modeling consist of time-invariant statistical methods that are able, to a degree, to mimic selected human hallmarks. Artificial intelligence frameworks must first be able to learn (training phase) a pattern and then produce models that are able to recognize the pattern in a prospective setting (testing phase).

Success using artificial neural networks (ANNs), one of the major classes of artificial intelligence, has been achieved in learning and reproducing critical elements from the fields of speech pathology and handwriting recognition, both of which require complex recognition. Each node on a neural net indicates a function and, as such, refers to a transformation. In this context, a neural network itself carries no values without input data. In oncology, neural networks have also been used successfully although they have yet to be used prospectively (119–121).

Much criticism has arisen in recent years on the application of ANNs to prediction problems in oncology (122). Single hidden layer ANNs are universal function approximators, meaning that they can theoretically represent any function, which is defined by their topology and weighting values. This may lead to the fitting of implausible functions to datasets yielding uninterpretable and simply illogical results.

##### Feed Forward Neural Network

**Figure 2:**
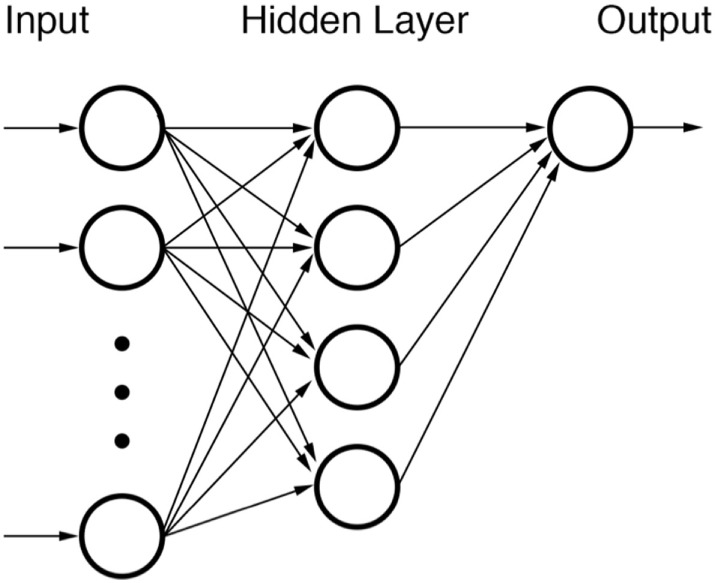
**Diagram of a feed forward neural network (FFNN)**. None of the nodes within the network are recursive.

Feed Forward Neural Networks (FFNNs) (Figure 2) do not include any recurring nodal inputs (“memories”) and are used frequently in basic pattern recognition problems. FFNNs are fully defined by their architecture such that arrangements of nodes into different topologies can induce different system responses. The user decides what topology to employ for a given FFNN during the training phase although some have demonstrated the feasibility of using separate optimization algorithms to optimize network architecture itself (123–125). In radiation oncology, attempts have previously been made to utilize FFNNs for their ability to classify highly non-linear data (126, 127).

Each node of a FFNN represents a function with one or more inputs. Inputs from previous nodes are transformed according to an activation function. Examples of commonly used activation functions are logit or probit functions. Other functions, such as the radial basis function (RBF), can also be used if data are suspected of originating from a specific type of distribution. After transformation by activator functions, outputs from nodes are stratified by weights. Such weights are the elements of the FFNN that are trained when building a FFNN. Training of nodal weights is relatively straightforward albeit time-consuming. The *delta rule* can be used via back-propagation to adjust node input and output weights until classification performance is optimized. Datasets for training can be used all at once (batch training) or can be segregated into pattern-based subgroups (sequential training).

One shortcoming of FFNNs is that extraction of relationship data from within them can be notoriously difficult, if not, impossible. Information for final output nodes is reliant on previous inputs and outputs and, therefore, can become extremely mathematically complicated. This disadvantage is somewhat of a trade-off given that the only time-intensive procedure is training of node weights, after which the network can be used for real-time classification. Validated FFNNs are, therefore, indeed amenable to clinical implementation.

At least one group has demonstrated the applicability of ANNs in predicting late RB and in fact demonstrated improved prediction performance over and above that of regression-based approaches (123). Their findings exploited a genetic algorithm for optimizing inputs into their neural network and, furthermore, leveraged multiple cross-validation phases.

##### Generalized Regression Neural Network

In contrast to the FFNN, the generalized regression neural network (GRNN) is a probabilistic neural network developed in 1991 and can, overall, be thought of as a best-fit estimator (128). The technique generalizes canonical regression by not being limited to a specific function (e.g., in linear regression) but instead expresses an empirical regression function as a probability density determined using a technique known as *Parzen window estimation*. To accomplish this, the technique utilizes the joint probability of the input vector(s) and the outcomes to calculate conditional probabilities and expected values. These values are used to estimate the generalized regression of the outcomes onto the input data. The joint probability of the input vectors and outcomes can be estimated via non-parametric estimators if not known outright.

In the context of training, the advantage of GRNNs is that they avoid having to backpropagate error to fine tune nodal weights, which is computationally expensive and time-consuming. Back-propagation is mitigated by dealing with probability distributions rather than discrete raw input data. This means that one-pass of the framework with training data is sufficient to estimate parameter weights. Previous work by our group has shown that GRNNs can outperform FFNNs when it comes to prospective applications in radiation oncology (129). This is likely a result of the probabilistic nature of nature and/or biological variables across a patient cohort.

##### Kernel-Based Methods

**Figure 3:**
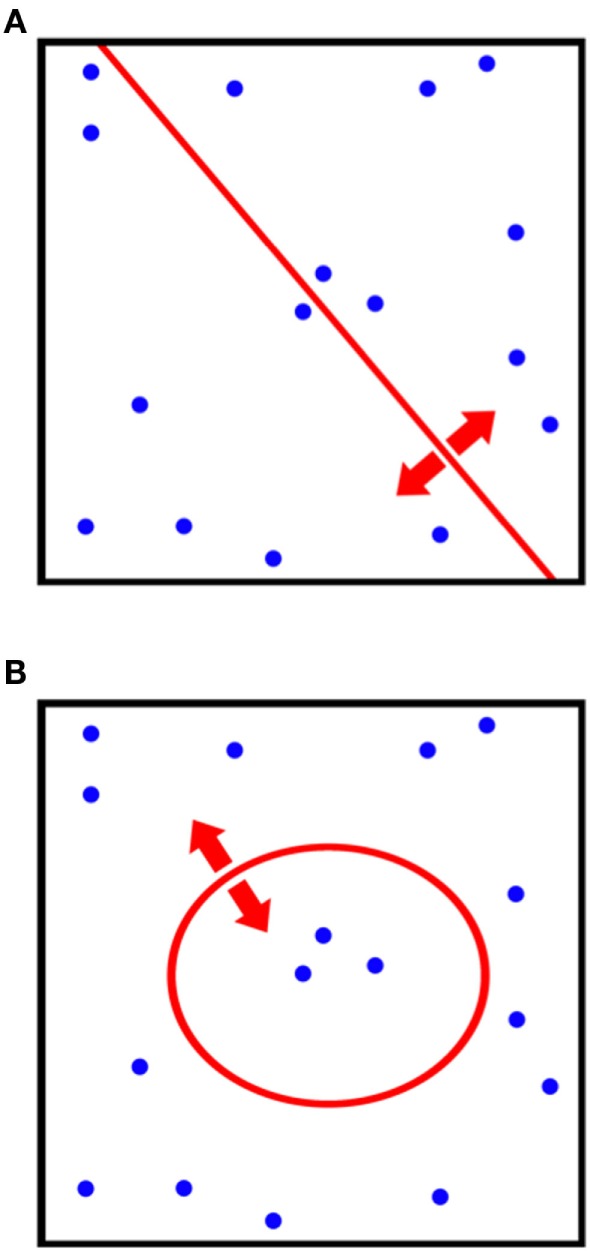
**Comparison of the differences in classification procedures of (A) canonical principle component analysis (PCA) and (B) kernel-PCA**. By utilizing a kernel transformation, non-linear thresholds can be used to separate and classify data.

Kernel-based approaches to classification problems are based on clustering data according to non-linear combinations of variables (such as hyperplanes) in order to separate data. Oncology data are oftentimes highly non-linear, which gives motivation to explore the application of such a technique. Kernel methods seek to maximize distances between clusters that have undergone non-linear transformations. In this sense, the technique is a non-linear analog of Fischer’s linear discriminant (FLD) analysis and principle component analysis (PCA) (Figure 3).

The most prominent member of the kernel-based learning family is the support vector machine (SVM). SVMs utilize support vectors that are formulated according to the most difficult to separate data and, therefore, are relied upon by the method in order to select an optimum classifier. By formulating the distance maximization problem between support vectors as a quadratic programing problem, a computationally efficient SVM formulation can be described by the following prediction function:

(15) $$f\left(X\right)=\sum_{i=1}^{n_{s}}\alpha_{i}y_{i}K\left(S_{i},X\right)+\alpha_{o}$$

where the number of support vectors is given by *n*_s_, *K* is the kernel transformation, and α_i_ is the coefficient determined by quadratic programing. Further details on kernel-based methods can be found readily in the literature (130–132) or in our previous work (133).

##### Systems Biology Approach

The concept of an integrated systems approach is that of understanding a given problem in terms of all of its components together, i.e., taking a “system-wide” view. This can be contrasted with a reductionist approach whereby each component of a system is looked at separately. A given system can be thought of having four principal components: structure (network topology), dynamics (time evolution of system), control (response and regulatory systems), and design (operational parameters or rules) (134). In applying a systems approach to biological and physiological systems, much insight could be gained that would otherwise be extremely challenging to extract using phenomenological models (135–137).

The biological effects induced by ionizing radiation are initiated at the atomic level in the form of free radical reactions. Free radical interactions occur rapidly and induce cascades of molecular responses, such as inflammation, which ultimately lead to recruitment of a variety of different molecular factors (138). Ultimately, over time, cellular responses can manifest themselves as clinical effects that are recorded as treatment outcomes, including toxicities. Much mechanistic insight and predictive power would be derived from a model that is able to combine these different organizational levels and related biophysical properties. Unfortunately, however, such a model requires expansive radiobiological knowledge spanning very different time and length scales, thus making the problem inherently complex.

**Figure 4:**
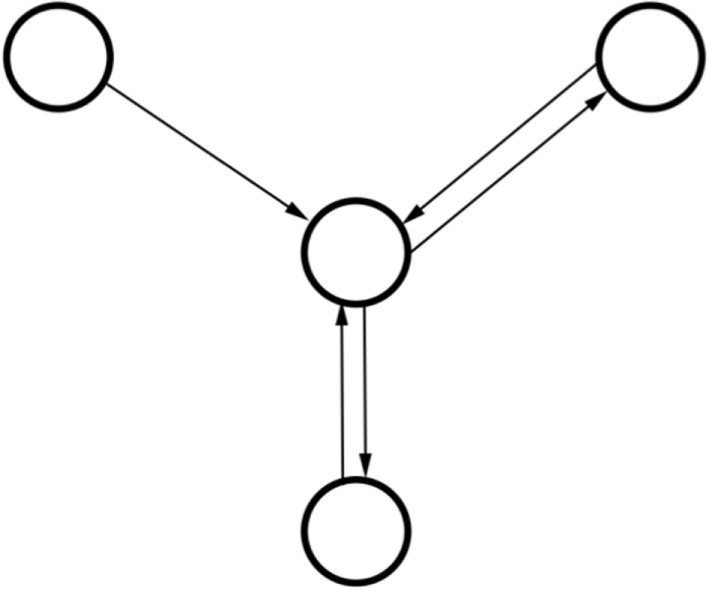
**Schematic diagram of relationships in a three-node graphical model with two recursive relationships**. Note how the model does not have a singular output as the outputs from the middle node are used as inputs. In contrast to artificial neural networks (ANNs), graphical models can take into account how variables are related via such conditional dependences.

In a related category, graphical models have been shown to be of use in radiation oncology as they can capture complex relationships between relevant factors and inter-dependencies between variables (Figure 4) (139, 140). Graphical models differ from aforementioned neural nets in that each random variable is represented by a node within the system and forms part of an intricately connected web. The web simulates conditional probability relationships making this class of algorithm classified as a structured prediction technique as oppose to clustering or regression (discussed previously). In previous work, our group has shown good classification performance using a graphical Bayesian network in predicting radiation-induced pneumonitis (116).

## Model Order Estimation

### Resampling Techniques

Frameworks that exploit resampling can be used to make estimates of model orders, parameters, or errors. In all cases, resampling requires that a dataset is repeatedly sampled with replacement in order to form many smaller, derived datasets. After several iterations, testing models on the derived datasets provides estimates on parameters of interest without requiring knowledge of the underlying distribution, which is a major benefit when little is known about the mechanics of the variable(s) of interest. Below, we discuss the two most commonly employed resampling techniques: jackknifing and bootstrapping.

#### Jackknifing

The jackknifing approach to parameter estimation entails systematically leaving out each of *N* samples and training *N* models on each of the *N − 1* remaining data points. The set of models trained on *N − 1* data points are then tested on the singular left-out data point one at a time. Analysis of the resulting *N* testing scores offers insight into how robust the model is under conditions of singular missing or inaccurate data (141, 142). Jackknifing is an approximation to the more labor-intensive, though robust, bootstrap technique.

#### Bootstrapping

“Bootstraps” are created from a given dataset by randomly resampling a given dataset (with replacement). Estimation of parameters or errors from each of the respective subsamples (a process known as bagging) can then be performed and averaged to yield an average value. The method is simple and not limited to specific classes of parameters and is, therefore, an extremely flexible technique (116, 118).

Bootstrapping is often used in cases where analytical error estimation is unfeasible. One shortcoming of bootstrap resampling is that it assumes independence of data points. In the context of radiotherapy outcomes, each set of data points originates from a specific patient and so this is often not an issue.

### Information Theory Approaches

Theoretical approaches to order estimation based on concepts borrowed from information theory can be used as alternatives to resampling techniques. These methods serve as tools to help identify which out of a finite number of models explain outcome data best. Therefore, the two methods discussed herein only indicate relative measures of fit and do not give any information on the quality of any model in an absolute sense (143).

#### Akaike Information Criteria

The Akaike information criteria (AIC) is based on the principle of good-of-fit and penalizes models that over- or under-fit data (144). The approach utilizes the Kullback–Leibler (K–L) distance, which quantifies the difference between two probability distributions. The K–L distance is used to estimate divergence of potential models from their true sampling distributions. The AIC approach furthermore rewards the models with the lowest value of the AIC parameter, which is calculated by considering the likelihood (*L*) of a particular model to explain outcome data (Eq. 16).

(16) AIC=2k−ln(L)

where *k* is the number of parameters in the model. In order to find the optimal AIC for a given set of models, the equation is minimized via maximizing the log-likelihood term on the right-hand side. The additional term *2k* is a penalty factor that penalizes over-fitting of data with increased number of variables. One shortcoming of AIC is that it can fail when large numbers of models are under examination due to the multiple comparison dilemma.

#### Bayesian Information Criteria

The Bayesian information criteria (BIC) is a closely related concept to AIC and can similarly provide information as to which out of many models bests explains a given set of data best (145). The BIC is based on Bayesian inference and is formally given by:

(17) BIC=k⋅ln(n)−2⋅ln(L)

where *L* is the maximum of the likelihood function, *k* is the number of parameters in the model, and *n* is the number of data points i.e., sample size. Threshold values for BIC that decidedly indicate whether a particular model should be discarded have been composed and tested by Kass and Raftery (p. 777) (146).

In comparison to the AIC, the BIC has a larger penalty term *k* ln*(n)* and, thus, penalize over-fitting more than does the AIC. As a result, BIC prefers models with fewer parameters than those chosen by AIC. The BIC also suffers when *k* is large due to the high-dimensionality problem of identifying variables that fit by chance.

## Evaluation of Model Performance

There exist numerous methods in literature to evaluate the ability of a given model to classify data in a prospective sense. Oftentimes, frameworks will employ more than one validation technique in order to explore the shortcomings of outputted models.

### Validation Coefficients and Metrics

Metrics and coefficients are the most readily available tools for calculating the prediction or classification performance of outcome models. Their simplicity is amenable to quick understanding of model behavior and, when several are used together, can yield insightful information.

The linear Pearson’s correlation is an example of a non-parametric coefficient that is used frequently for estimating the linearity of a relationship between two variables. More often employed in outcome models is the Spearman Rank Coefficient, which does not assume linearity and instead yields an estimate on the direction of trend between two parameters.

Alternatively, receiver-operating characteristic (ROC) values can be summed from ROC plots to readily convey classification performance alongside sensitivity and specificity for the desired classification cutoff value.

### Cross-Validation by Resampling

Resampling with replacement can be used to quantify the classification performance of models as well as estimate confidence intervals on model performance or provide estimate son the error of classification statistics. In our experience, leave-one-out cross-validation (LOOCV) on finalized models serves as an excellent method to quickly estimate how robust a given model is without having to rely on more computationally expensive methods, such as bootstrapping.

## Model Performance Visualization

### Octile Plots

**Figure 5:**
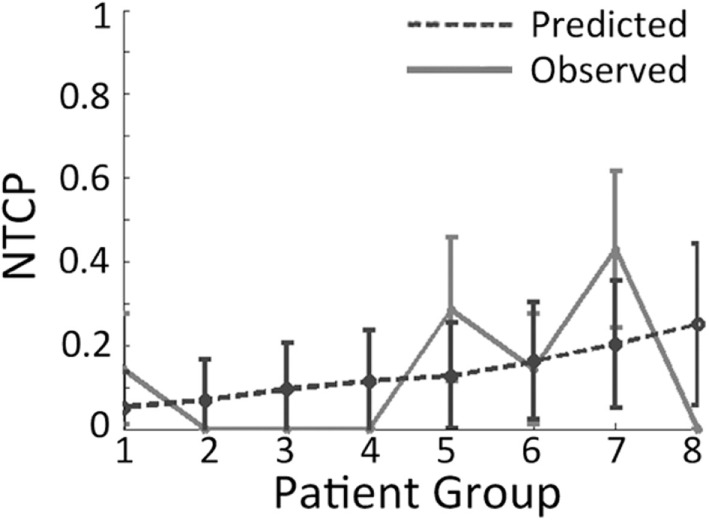
**Example of an octile plot demonstrating how patients are sub-divided into eight groups and then stratified according to the risk given by the model of interest**.

Plots whereby outcomes are split into eight groups (octiles) are called octile plots (Figure 5). By considering and plotting both the predicted and observed outcomes, the plot provides a two-dimensional method to visually assess model fit. Furthermore, octile plots allow the reader to gage how the overall model output varies with increasing magnitude of input parameters.

### Receiver-Operating Characteristic Plot

As discussed previously, the ROC plot can provide a method for visually assessing model performance. Although the AUC parameter derived from the ROC plot is reported more often, the ROC plot itself is also useful as it provides information on how the sensitivity varies with the specificity for different threshold cut-offs. In our work, we often use ROC plots alongside AUCs and correlation coefficients to gain a full understanding of how a particular model behaves under conditions of cross-validation (60).

### Vector Biplots

Biplots display vectors that are constructed and presented alongside PCA-derived information and patient-specific NTCP. In the context of model visualization, vector biplots provide a rough estimate of which variables are able to explain the data – this is generally accomplished in two dimensions to aid visualizing the model on paper.

### PCA and Kernel-PCA

**Figure 6:**
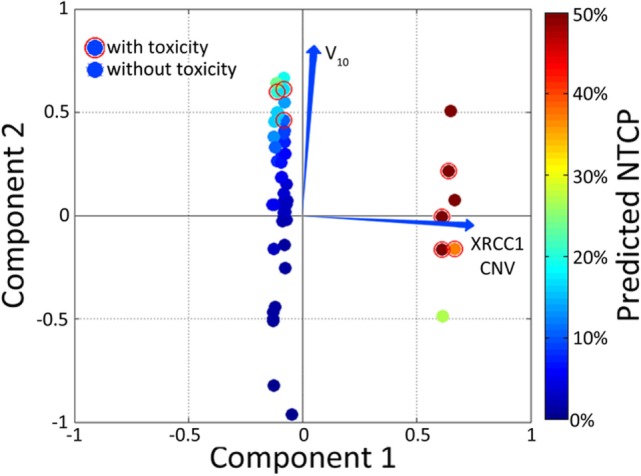
**Example of a color-washed vector biplot**. The cardinal plane represents the vector biplot relating to the magnitude of the contribution of the variables contained within the model. The axes on which the vector biplot is shown are derived via principle component analysis (PCA). Dummy patients with toxicity were circled with empty red circles and color-washed according to NTCP values generated via the model.

Principle component analysis is useful as a method to reduce the dimensionality of data into either two- or three-dimensions to facilitate performance visualization. The trade-off in using PCA for outcome modeling is that the relationships between inputs and outcomes are highly non-linear and, therefore, true relationships may not be adequately captured by the technique. Alternatively, the previously described Kernel-PCA technique can be employed to visualize data by improving separation between clusters. Vector biplots, two- and three-dimensional kPCA plots can indeed be used together in order to provide easy-to-interpret heat maps colorized by estimated patient-specific risk (Figure 6) (147).

## Controversies

The number of variables in a big data analysis of outcome models can accumulate quickly, especially if considering many biological factors. Several of the modeling techniques presented herein fail to take into account or mitigate the dilemma of multiple comparisons of chance correlations. Therefore, in all aspects of modeling, independent validation phases should be integrated into frameworks that aspire to produce clinically relevant results. Furthermore, although internal cross-validation techniques do provide excellent estimates of model robustness, their usefulness is limited if training samples are not retrieved from independent sources.

Radiotherapy outcomes are complex pathological manifestations of the biophysical effects of ionizing radiation on the human body. Therefore, models attempting to delineate such phenomena should endeavor to incorporate as many different types as possible.

By definition, big data requires the ability to work with extremely large multi-dimensional datasets, which requires dedicated infrastructure to support access as well as high performance capabilities to facilitate efficient data exploration and modeling. Such investments require capital expenditure and training in addition to the formation of data-sharing and privacy agreements between institutions (148).

Once meaningful analytics can readily be extracted from multicenter databases, scientists and physicians are faced with the dilemma of determining how their predictive models should be used to maximize the TCP/NTCP ratio. No doubt, the aforementioned biological and clinical risk factors that predispose a prostate cancer patient to radiation toxicity can be quantified before therapy and used to guide initial radiation treatment planning but such an approach ignores such factors as risk to long-term quality of life, intra-treatment imaging data, physiological changes during therapy, and symptoms the patient may develop during and shortly after therapy.

## Software Tools

Many independently verified platforms exist for data-mining and analytics exploration, several of which are listed below with brief explanations of their scope and limitations. Further details on the use and QA of such software can be found in AAPM Task Group Report #166 (149).

### BIOlogical Evaluation of PLANs (BIOPLAN)

Bioplan is a user-friendly software developed in the United Kingdom that allows an absorbed dose treatment plan to be converted into its likely biological effect (150). It provides a variety of tools, including DVH subtraction, and is able to calculate NTCP values according to previously described LKB and binomial-based models.

### Computational Environment for Radiotherapy Research

Computational environment for radiotherapy research (CERR) is an open-source computational environment that facilitates the conversion of treatment plan data into MATLAB (151). The software allows for either retrospective or experimental treatment planning and can read-in CT data as well as associated contours. In the past, our group has used CERR in investigating both GU and GI toxicities in prostate cancer. CERR can also be used to estimate the contribution of joint-contours, such as rectal margin overlap with the PTV, to toxicities.

### Dose Response Explorer System

Dose response explorer system (DREES) is an open-source data-mining tool for exploring dose–response relationships (142). Using a built in subroutine called CERR+, the program imports patient data from CERR. DREES provides a suite of tools for either NTCP or TCP modeling of outcomes without restriction as to the site or population size. The program includes a GUI interface within MATLAB in order to simplify usability. Examples of the functions contained with DREES include logistic regression, LKB modeling, actuarial statistics, bootstrap validation, Kaplan–Meier survival analysis, nomograms, boxplots, and has the functionality for interfacing with SVMs. One of the major advantages of using DREES is it that it freely distributed, as is CERR, and is consistently updated, making it flexible and adaptable.

## Future Trends

### Implications of Charged Particle Therapy

The use of charged particle therapy in treating prostate cancer has become more mainstream over the past decade, mainly in the United States (152). Unfortunately, comparable outcome data from a treatment delivery technology point of view on the use of proton therapy vs. photon therapy for prostate cancer (to an even lesser extent carbon ion therapy) are not yet readily available. Unlike the mainstream adoption of RBE = 1.1 for proton therapy (153), the RBE debate for charged particles continues, which makes outcomes comparison to photons difficult for heavier charged particles and prospective outcomes prediction impossible.

Studies published using modulated proton techniques report that GI/GU late effects post-RT are either unchanged (154) or reduced (155–157). In the case of carbon ions, extensive long-term biochemical outcomes are not yet available and so does the interpretation of the efficacy of such treatments remains difficult. In terms of late radiation-induced ED, at least one study has shown no significant upregulation using protons (158).

The impact of charged particle therapy plans on outcome modeling is that thresholds, such as those proposed by QUANTEC, will likely need to be adjusted given the differences in dose distribution and biological response relative to photon-based therapy (159, 160).

### Advanced Methods in Machine Learning

Examples of more complex modeling techniques include the use of restricted Boltzmann machines (RBMs), which are energy-based multilayered graphical models that estimate the joint probability distribution between inputs and outcomes using one or more binary stochastic hidden layers (161–163). The concept of an RBM was first proposed by Prof. Geoffrey Hinton from the University of Toronto in 2006 as a method to efficiently train and learn a constrained version of a Boltzmann machine (164). RBMs as applied to oncology have shown promise in making accurate predictions, however, they remain relatively poorly disseminated techniques and their implementation to date has been limited.

Multilayered networks, such as RBM or convolutional neural network, can perform *deep learning* in that data are passed through more than one layers of machine-learning modules that combine to form a framework capable of processing highly complex patterns. More specifically, deep learning strategies attempt to model high-level abstractions, such as the recognition of three-dimensional objects, in order to label and classify. This may prove extremely useful in the case of radiation-induced biological effects given the physical understanding that such phenomena manifest after cascading effects at the atomic, molecular, and then cellular levels. Previously, modeling strategies involving deep learning in the form of deep belief networks (DBNs) have been applied to data in oncology via multilayered RBMs, which are known as deep Boltzmann machines (DBMs) (165, 166). Such applications are, for instance, infrequent but may become more frequent as techniques are disseminated and refined specifically for the purposes of radiation oncology.

## Conclusion

Prostate cancer is one of the most commonly diagnosed cancers across the globe and radiation therapy is a primary modality in treating such cases. Prostate cancer is a heterogeneous disease at several levels (pathological, molecular/genetic, and clinical) and, despite technical improvements, there is still a significant risk of cancer recurrence after therapy. Treatment efficacy for localized prostate cancer has increased greatly in recent years; however, few efforts have been aimed at developing and testing personalized predictive metrics, namely those identified through the rapidly advancing field of big data analytics using machine learning and artificial intelligence. Such analytics would allow prostate radiotherapy regimens to be further tailored to the individual and generate treatment plans that are functions of not only dose–volume metrics but also individual’s clinical risk factors and biological parameters.

Given the apparent complexity of physiological response to ionizing radiation, it is likely that systems-based approaches will play a larger role in radiotherapy outcome modeling in the future. Although regression-based techniques have yielded success in certain cases, their cross-validated prediction performance appears to be generally limited, likely due to their inability to capture higher order interactions between biophysical processes.

A chief limitation in modern outcome modeling projects is the difficulty in pooling sets of data from multiple institutions. In medicine, this is principally due to privacy and security concerns. If, however, proper data-sharing protocols can be put in place, big data analytics may provide a significant boost to data-driven outcome models owing not only to larger datasets but also to the ability to more readily perform independent validation.

## Author Contributions

The initial conception of the review article was proposed by IN and the initial draft was compiled by JC. Multiple rounds of peer editing by both JC and IN then took place until LS was contacted at the request of the editors in order to review the manuscript for clinical input. All figures were selected and compiled together by JC, LS, and IN.

## Conflict of Interest Statement

The authors declare that the research was conducted in the absence of any commercial or financial relationships that could be construed as a potential conflict of interest.
